# Genome-wide analysis of IQD proteins and ectopic expression of watermelon ClIQD24 in tomato suggests its important role in regulating fruit shape

**DOI:** 10.3389/fgene.2022.993218

**Published:** 2022-09-14

**Authors:** Junling Dou, Shixiang Duan, Muhammad Jawad Umer, Kuixi Xie, Yinping Wang, Qishuai Kang, Sen Yang, Luming Yang, Dongming Liu, Lifeng Liu, Fengli Zhao

**Affiliations:** ^1^ College of Horticulture, Henan Agricultural University, Zhengzhou, China; ^2^ State Key Laboratory of Cotton Biology, Cotton Institute of the Chinese Academy of Agricultural Sciences, Anyang, China; ^3^ Zhengzhou Fruit Research Institute, Chinese Academy of Agricultural Sciences, Zhengzhou, China; ^4^ College of Horticulture, Nanjing Agricultural University, Nanjing, China

**Keywords:** watermelon, IQD family, ClIQD24, ectopic expression, fruit shape

## Abstract

The plant-specific IQ67 domain (IQD) is the largest class of calmodulin targets found in plants, and plays an important role in many biological processes, especially fruit development processes. However, the functional role of IQD proteins in the development of watermelon (Citrullus lanatus) shape remains unknown, as the IQD protein family in watermelon has not been systematically characterized. Herein, we elucidated the gene structures, chromosomal locations, evolutionary divergence, and functions of 35 IQD genes in the watermelon genome. The transcript profiles and quantitative real-time PCR analysis at different stages of fruit development showed that the ClIQD24 gene was highly expressed on 0 days after pollination. Furthermore, we found that the ectopic overexpression of ClIQD24 promoted tomato fruit elongation, thereby revealing the significance of ClIQD24 in the progression of watermelon shape. Our study will serve as a reference for further investigations on the molecular mechanisms underlying watermelon fruit shape formation.

## Introduction

Plants activate a series of signal transduction pathways to coordinate physiological and metabolic responses to adapt to adverse environmental conditions. Calcium (Ca^2+^) is a secondary messenger, and spatial and transient changes in cytoplasmic Ca^2+^ levels are triggered by abiotic stress and perceived by the Ca^2+^ sensor (Aldon et al., 2018). Calmodulin (CaM) is one of the most intensively studied Ca^2+^ sensors that mediate Ca^2+^ signal transduction into cellular responses through the modulation of various target proteins (Yuan et al., 2022). The plant-specific IQ67 domain (IQD) protein is the largest class of CaM targets and has been identified in many plants (Rehman et al., 2021).

IQD proteins were originally identified in Arabidopsis thaliana. They are a type of CaM binding protein, which has been identified in many species from lower bryophytes to higher angiosperms (Abel et al., 2005). IQD proteins are characterized by the presence of a conserved IQD consisting of 67 amino acid residues, which recruits CaM and serves as a Ca^2+^ sensor (Rehman et al., 2021). Generally, the IQD is composed of 1 - 3 tandem repeats of CaM-binding IQ motifs, each containing two conserved isoleucine (I) and glutamine (Q) amino acid residues. Currently, two types of IQDs have been found in plants, the Ca^2+^-independent IQ motif (IQxxxRGxxxR and I/L/VQxxxRxxxxR/K) and Ca^2+^-dependent IQ motifs (1-5-10 and 1-8-14 motifs) (Wu et al., 2011). Moreover, IQD proteins have been annotated in several plant species, including tomato, cotton, and potato (Mei et al., 2021).

With the development of sequencing technology, IQD proteins have been found to play vital roles in various cellular processes, including host defense, cell shaping, and drought resistance (Yuan et al., 2019; Liu et al., 2020). The AtIQD1 gene from Arabidopsis thaliana was found to regulate glucosinolates (a class of defense-related metabolites). It has been reported that the overexpression of AtIQD1 can stimulate glucosinolate accumulation and plant defenses in transgenic Arabidopsis (Levy et al., 2005). Additionally, AtIQD11, AtIQD14, and AtIQD16 regulate plant growth and cell shape through unknown mechanisms that may be related to the CaM-dependent Ca^2+^ signaling pathway (Bürstenbinder et al., 2017). Two other IQD genes from Arabidopsis and rice have also been found to regulate plant growth and development (Duan et al., 2017; Sugiyama et al., 2017). Collectively, these findings show that IQD genes play important roles in plant growth and development. IQD protein interacts with microtubules to regulate the progression of organ shape (Lazzaro et al., 2018). Moreover, IQD proteins serve as scaffolding proteins and have been linked to Ca^2+^ signals in some organelles, regulating plant growth (Burstenbinder et al., 2017). In addition, the IQD protein IQD1 reportedly interacted with KLCR1 and CaM, which induced kinesin and Ca^2+^ secondary messenger signaling, thereby participating in cargo transport (Kong et al., 2015).

IQD protein families have been comprehensively analyzed in many crops including Arabidopsis, Solanum lycopersicum, Oryza sativa, Glycine max, Phyllostachys heterocycla, Brachypodium distachyon, and Populus trichocarpa (Zhou et al., 2010; Filiz et al., 2013; Huang et al., 2013; Feng et al., 2014; Ma et al., 2014; Wu et al., 2016). Subsequently, the functions of IQD proteins were investigated and found to play important roles in many pathways, including plant defense responses, the regulation of microtubules organization, plant growth and development, and cell shape (Burstenbinder et al., 2017). In Arabidopsis, except for the function of plant defenses, AtIQD1 also resulted in glucosinolate accumulation, which enhanced herbivory resistance (Levy et al., 2005). AtIQD22 has been shown to negatively regulate plant responses to GA (gibberellin) (Zentella et al., 2007). In tomato, a 24 kb chromosome fragment repeat increased SlSUN12 expression (a homologous gene of IQD) and copy numbers, which led to fruit elongation (Xiao et al., 2008). SlSUN24 regulates tomato seed germination by participating in the ABA signaling pathway (Bi et al., 2018). CsSUN, a homolog of tomato fruit shape gene SUN, regulates cucumber fruit shape (Pan et al., 2017). In soybean, 24 GmIQD III genes were regulated by MeJA (methyl jasmonate) stress (Feng et al., 2014). Similarly in Populus trichocarpa, the expression of 12 selected IQD members was regulated by PEG (polyethylene glycol electrolyte solution) and MeJA treatments (Ma et al., 2014).

As a member of Cucurbitaceae, watermelon (Citrullus lanatus) is grown widely and is very popular with consumers all over the world. It has 11 chromosomes (2n = 2X = 22) with a 425 Mb reference genome size approximately. The watermelon reference genome “97103” v1 was sequenced and released in 2013 (Guo et al., 2013). Subsequently, the “Charleston Gray” reference genome and “97103” v2 were also been released (Wu et al., 2019). Although the genome sequencing of watermelon has been completed, only a few studies have reported the IQD proteins in watermelon. Only the ClFS1 gene, which belongs to the IQD protein family, had been identified to regulate fruit shape. The 159 bp deletion in the ClFS1 gene was responsible for elongated fruit (Dou et al., 2018). Additionally, the watermelon IQD protein family has not been systematically analyzed. The function of IQD during watermelon development was unknown. In this study, we elucidated the gene structures, chromosomal locations, evolutionary divergence, and functions of 35 IQD genes in the watermelon genome. Furthermore, the transcript profile of the 35 ClIQD genes was analyzed at 0 DAP (days after pollination), 7 DAP, and 14 DAP. We found that ClIQD24 was highly expressed only in ovary development. The ectopic expression of ClIQD24 in tomato promoted fruit elongation, indicating that ClIQD24 proteins play important roles in watermelon fruit development. The findings of this study will serve as a reference for future investigations on the function of ClIQD genes in watermelon.

## Materials and methods

### Identification of the ClIQD proteins in watermelon

To identify ClIQD genes in the watermelon genome, two database searches were performed. First, we retrieved 33 IQD protein sequences from TAIR (Arabidopsis Information Resource, https://www.arabidopsis.org/). Then the 33 IQD proteins were used as queries to perform a BLAST-P search against the NCBI database (http://www.ncbi.nlm.nih.gov). Secondly, all selected proteins were used as queries to perform BLAST-P searches in the Watermelon Genome Database (http://cucurbitgenomics.org/). The SMART online database and the Pfam database (http://pfam.xfam.org/search/) were used to confirm that all putative non-redundant sequences contained the canonical consensus. Finally, we identified 35 IQD proteins in the watermelon genome. Details of the watermelon ClIQD sequences, including the number of exons, open reading frame (ORF) lengths, amino acid sequence lengths, chromosome location, and isoelectric point (PI), were obtained from the Expasy Proteomics Server (http://web.expasy.org/compute_pi/) (Supplementary Table S1).

### Bioinformatics analysis of the ClIQDs in watermelon

The ClIQD genes were renamed based on the gene IDs from chromosomal 0 to chromosomal 11. According to the chromosomal localization data on the chromosome, the ClIQD genes were mapped to the chromosomes using MapInspect software (http://mapinspect.software.informer.com/1.0/) (Zhang et al., 2013). Exon-intron organization of the watermelon IQD genes was illustrated using the Gene Structure Display Server (GSDS2.0, http://gsds.cbi.pku.edu.cn/) by comparing genomic sequences with their corresponding coding DNA sequence (CDS). The online MEME program (http://meme.nbcr.net/meme/cgi-bin/meme.cgi) was used to analyze conserved motifs of the watermelon IQD proteins (Bailey and Elkan, 1955). Pfam was used for motif annotation with the following parameters: optimum motif width 6–100 residues, the maximum number of motifs 10 (based on many times comparisons and settings, Supplementary Table S2).

According to the available information, 33, 28, and 33 IQD amino acid sequences of Arabidopsis thaliana, rice, and tomato were retrieved from their reference genomes, respectively (Huang et al., 2013; Ma et al., 2014). Information on these amino acids is presented in Supplementary Table S3. According to the sequence alignments, a phylogenetic tree was constructed using MEGA7.0 software (Shi et al., 2016). The NJ (neighbor-joining) method was used to generate a model using Poisson’s correction, 1000 bootstrap replicates, and pairwise alignment (Wei et al., 2019).

### RNA-seq library construction, sequencing, and reads mapping

The watermelon accessions WM102 is a homozygous inbred line, that was manually self-pollinated for at least five generations in this study. All watermelons used in this study were sown in nursery trays, and the seedlings were transplanted to greenhouses at the Science and Education Park of Henan Agricultural University, Zhengzhou, China. To study the ClIQD gene expression differences during fruit development, fruit at different development periods (0 DAP, 7 DAP, 14 days DAP) were used for RNA-seq analysis (Biomarker Technologies Co. Ltd., Beijing, China).

Total RNA was extracted from fresh watermelon rind samples using the CTAB method. Samples (1.0 g) with three biological replicates were collected during each developmental stage (0 DAP, 7 DAP, 14 DAP). RNA concentrations and quality were determined and assayed using a NanoDrop 2000 (Thermo) and 1.5% agarose gel, respectively. The RNA-seq library was used to prepare the sample libraries based on the constructed flow path, and then the RNA was sequenced on an Illumina HiSeq 2000 platform. The raw sequencing data from the RNA-seq were used for subsequent analysis. Clean reads were obtained by filtering, contaminated sequences, and low-quality reads and then aligned to the watermelon reference genome “97103” v1 (Guo et al., 2013) according to HISAT2 (Kim et al., 2015). The transcript data were assembled using Stringtie software (Pertea et al., 2016). ClIQD expression was analyzed using the FPKM method (Mortazavi et al., 2008). A heatmap was generated using TBtools. The RNA-seq data (Liu et al., 2020) are available in the NCBI database (accession no. PRJNA549842) (Supplementary Table S4).

### Quantitative real-time PCR (qRT-PCR) analysis

To verify RNA-seq results, watermelon rind samples were collected at different developmental periods (0DAP, 7DAP, 14DAP) and used for RNA extraction to investigate the expression patterns of the ClIQD genes during fruit developmental. Total RNA was extracted using a plant RNA purification kit (Omega) following the manufacturer’s instructions. The RNA quantity and quality of RNA samples were determined and assayed using a NanoDrop 2000 (Thermo) and 1.5% agarose gel, respectively. The cDNA was synthesized using reverse transcriptase M-MLV (RNase H-) following the instructions of the manufacturer (Takara, Japan). The primers used for qRT-PCR were designed to amplify products ranging from 100–250 bp using Primer 5.0 online software. The Actin gene primer (Kong et al., 2015) and 35 ClIQD gene primers are listed in Supplementary Table S5.

The expression patterns of the ClIQD genes were evaluated by qRT-PCR using the system of LightCycler480 RT-PCR system (Roche). The PCR reaction system had a total volume of 20 μL, consisting of 10 μL 2× Master Mix (Roche), 1 μL each of 10× primers, and 100 ng of genomic cDNA. All amplifications were carried out on a LightCycler 480 High-Resolution Melting Master system. The PCR reaction was as follows: 94°C for 10 min; 45 cycles at 95°C for 15 s, 60°C for 20 s, 72°C for 25 s; 72°C for 10 min; High-Resolution Melting was performed under the following conditions: 95°C for 1 min, 40°C for 1 min, 65°C for 10 s, continuous 95°C. Normalized, temp-shifted melting curves from CyP2C2 amplicons carrying a sequence variation were evaluated using LightCycler 480 Gene Scanning Software. Experiments were performed in triplicate with three technical replicates. The relative expression pattern was performed and calculated using the 2^-△△CT^ method (Dou et al., 2018).

### Ectopic expression of ClIQD24 in tomato

To verify the regulation of ClIQD24 during watermelon fruit development, the ClIQD24 overexpression vector was constructed using the pRI101 vector. The pRI101 vector was cleaved at the restriction endonuclease cleavage site of, ScaI and XbaI, and then the amplified CDS sequence of the ClIQD24 gene was inserted into a linearized pRI101 vector. The primers used for vector construction are listed in Supplementary Tables S6. The overexpression vector ClIQD24-pRI101 was fused into Agrobacterium using the heat shock method and then transformed to tomato Micro-Tom (Sun et al., 2006; Ding et al., 2019). The Micro-Tom seeds were sterilized as follows: 75% ethanol for 20 s, rinsed with ddH_2_O 3 times, 10% sodium hypochlorite for 1–2 h, and dried on sterile filter paper. Transgenic positive plants were grown in a tissue culture room at 25°C, 75% relative humidity, and 250 μmol m^−2^ s ^−1^ light intensity under a 14/10 days/night photoperiod. Plants were grown in nutrient bowls until the plants had three leaves and one heart. We measured the fruit length (cm), width (cm), and fruit shape index during the fruit ripening period of overexpression-positive tomato plants. Each individual had five fruits measured as biological replicates.

### Prediction of miRNA targeting watermelon ClIQD24 gene

The sequence of ClIQD24 was used as candidate gene to identify possible miRNAs based on the psRNATarget database (https://www.zhaolab.org/psRNATarget/analysis?function=2. Accessed on 5 August 2022) with default parameters (Raza et al., 2021; Wang et al., 2022). Moreover, the interaction network between ClIQD24 and the identified miRNAs was bulided using the software Cytoscape (V3.8.2, https://cytoscape.org/download.html. Accessed on 3 August 2022).

## Results

### Identification and annotation of watermelon IQD genes

In the Arabidopsis genome, 33 IQD protein sequences are easily available on 8 chromosomes (TAIR, https://www.arabidopsis.org/) (Abel et al., 2005). The 33 Arabidopsis IQD proteins were used as query sequences to search watermelon IQD protein sequences in the NCBI database and Cucurbitaceae Genome Database (http://cucurbitgenomics.org/). Then, the multiple sequence alignment analysis was performed using the MEGA 7.0 software. Conserved domains of the candidate protein sequences were analyzed using SMART (http://smart.embl-heidelberg.de/smart/) and Pfam (http://pfam.xfam.org/search/) online software. Finally, 35 watermelon IQD genes were identified by deleting repetitive and incomplete domain sequences. The number of IQD genes identified in watermelon was slightly larger than the numbers previously reported in Arabidopsis (33), rice (28), and tomato (33) (Abel et al., 2005; Choi et al., 2005; Ma et al., 2014). To unify the naming of watermelon IQD genes, the 35 watermelon IQD genes were named ClIQD1 - ClIQD35 (Supplementary Tables S1, S2).

The physical and chemical properties such as the molecular weight, length, and isoelectric point (pI), of all ClIQD proteins were obtained by importing them into the EPasy site (Supplementary Table S1). The amino acid lengths of the ClIQD genes ranged from 64 to 1073 with an average length of 443 amino acids. The molecular weights ranged from 7.3 kD to 117.1 kD with an average weight of 49.6 kD. These parameters are similar to those in Arabidopsis and rice (Choi et al., 2005). In addition, most watermelon ClIQD proteins had relatively high pI and other important physical and chemical properties except the ClIQD32. The pI of ClIQD32 was 5.08 with a length of 1073 amino acids and a molecular weight of 117.1 kD (Supplementary Table S1).

### Phylogenetic analysis of IQD genes in watermelon, arabidopsis, rice, and tomato

**FIGURE 1:**
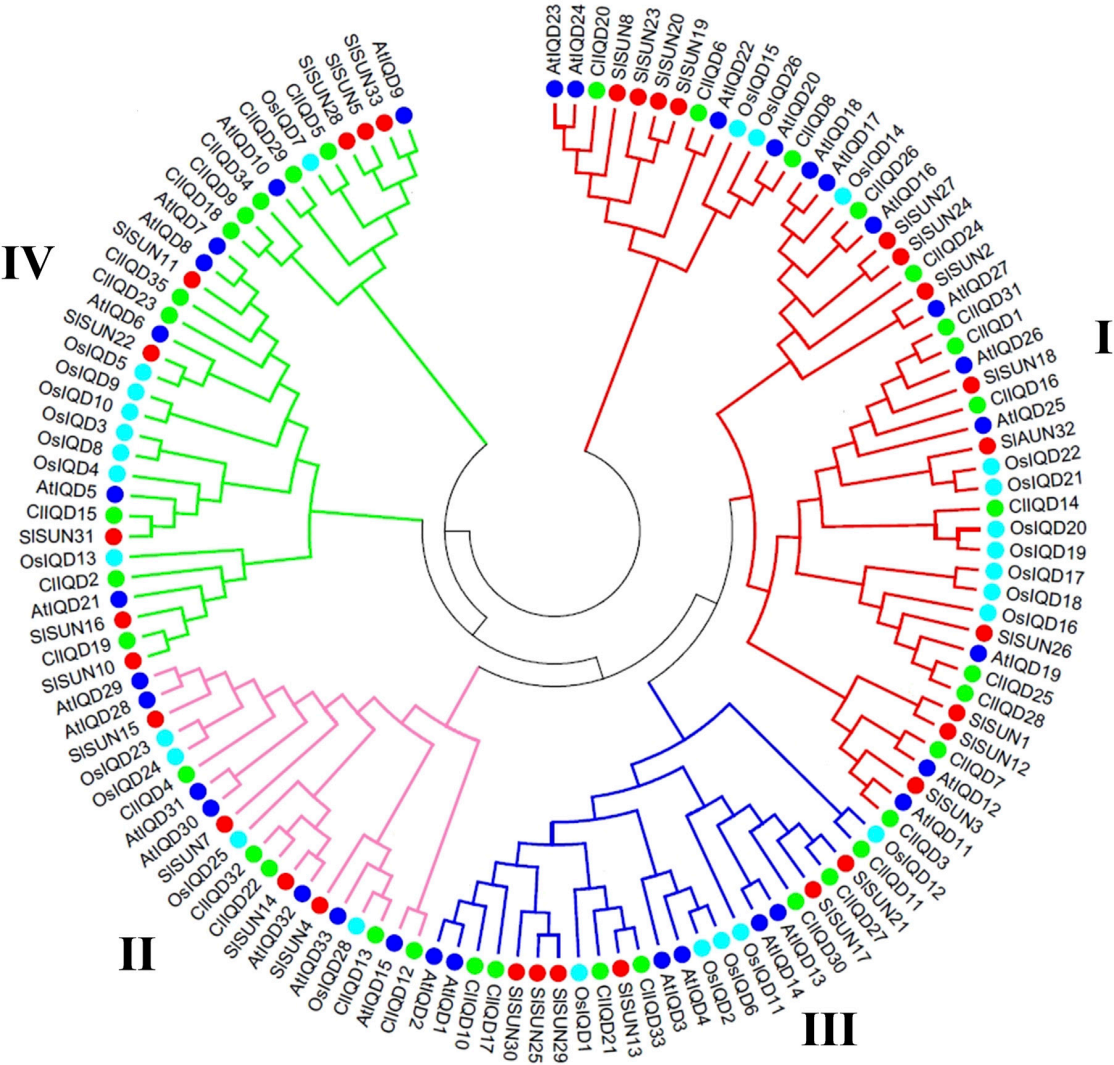
Phylogenetic relationships of Arabidopsis, rice, tomato, and watermelon IQD members. The full-length amino acid sequences of 129 IQD proteins were aligned using ClustalX 1.83. The phylogenetic tree was constructed using the neighbor-joining (NJ) method by MEGA 5.0. Each IQD class is indicated by the specific line color. Redline, class I; Pink line, class II; Blue line, class III; Green line, class IV.

To further investigate the evolutionary relationships and classify genes to obtain potential functions of the ClIQD proteins from previous studies, a total of 129 IQD genes containing 33 AtIQDs from Arabidopsis thaliana (Abel et al., 2005), 33 SlSUNs from tomato (Huang et al., 2013), 28 OsIQDs from rice (Choi et al., 2005) and 35 ClIQDs from watermelon were used to construct a phylogenetic tree. The full-length IQD amino acid sequences were aligned and the phylogenetic analysis was conducted using the neighbor-joining (NJ) method implemented in MEGA 7.0. The 129 IQD proteins were classified into four distinct subfamilies (group I, group II, group III, and group IV) (Figure 1). Among these four subfamilies, group I contained the largest number of IQD protein members (13 ClIQD proteins) from watermelon, while group II had the least 5 ClIQD proteins IQD classifications suggested that the ClIQDs may serve different functions in different groups.

The IQD members consisting of four different species in the same subfamily had a closer evolutionary relationship than different subfamilies consisting of the same species (Figure 1). Further analysis showed that dicotyledonous plants, including Arabidopsis thaliana, tomato, and watermelon, had more similar evolutionary relationships among the IQD domains. The IQD proteins’ evolutionary relationships between monocotyledonous (rice) and dicotyledonous plants (Arabidopsis thaliana, tomato, and watermelon) were not far, which was consistent with previous reports (Feng et al., 2014; Cai et al., 2016). Collectively, these findings showed that the evolutionary process of IQD proteins in watermelon was consistent with monocotyledonous and dicotyledonous plants.

### Chromosomal locations of the ClIQD genes

**FIGURE 2:**
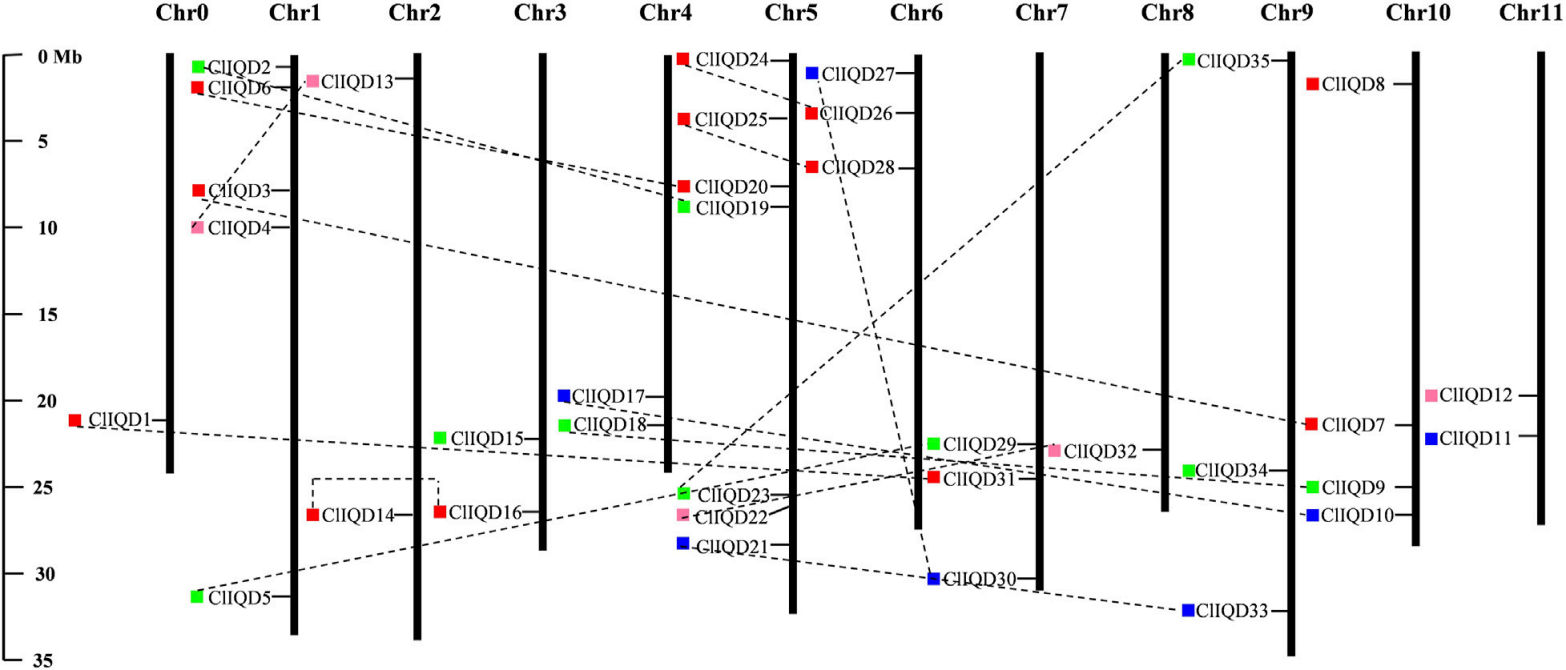
Chromosomal distribution and duplication events of watermelon IQD genes. The 35 ClIQD genes are widely mapped to the 11 watermelon chromosomes. Duplicated paralogous pairs of ClIQD genes in segmentally duplicated blocks are connected with black dashed lines. The chromosome number is located at the top of each vertical bar. The chromosomal position of each maize IQD gene is indicated by the gene name. Colored boxes to the left of the gene name represent the corresponding subfamily to which the gene belongs.

Based on the location information of ClIQD genes in the Cucurbitaceae Genome Database, the location information of 35 ClIQD genes was obtained and the genes were mapped to the 11 watermelon chromosomes using MapInspect chromosome positioning software. The physical location information and distribution of the ClIQD genes across the 11 chromosomes are shown in Figure 2. The 35 ClIQD genes were widely distributed across the 11 watermelon chromosomes, while the distribution within each chromosome was uneven. Seven ClIQD genes were distributed on chromosome 5, which was the greatest number of genes among the 11 chromosomes, while only one ClIQD gene was distributed on chromosome 2 and chromosome 8, respectively. Of these 35 ClIQD genes, five (ClIQD8, ClIQD11, ClIQD12, ClIQD15, ClIQD34) have a far relationship, other 30 ClIQD genes have homologous genes with each other (Figure 2). The location patterns of the ClIQD genes in watermelon were similar to OsIQD genes in rice but differed from AtIQD genes which were evenly distributed across the eight Arabidopsis chromosomes (Abel et al., 2005; Rodriguez et al., 2011).

### Conserved motif identification and gene structure analysis of the ClIQD genes

**FIGURE 3:**
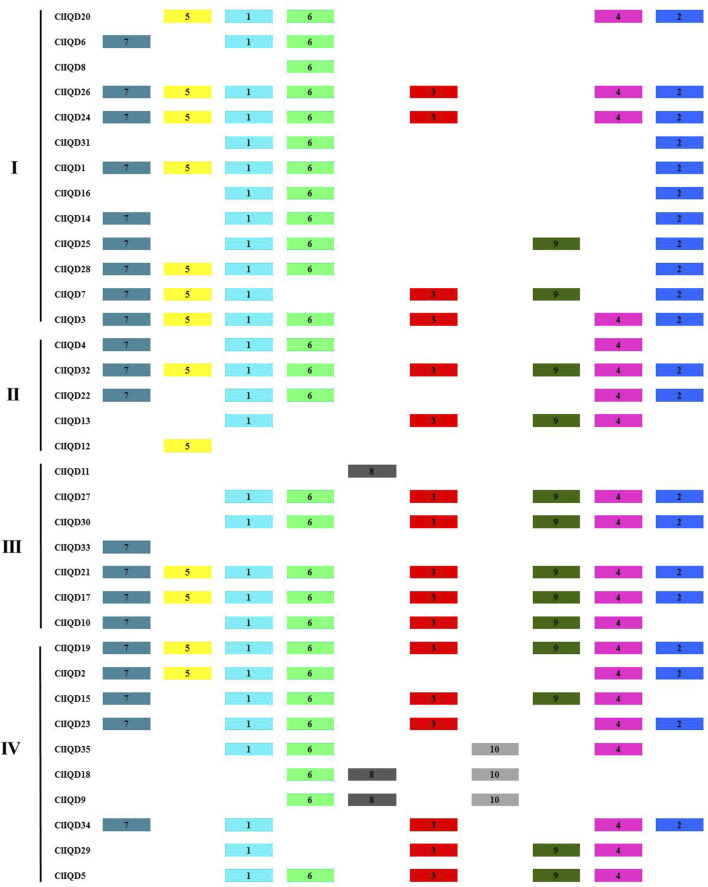
Motif patterns of the 10 conserved motifs in ClIQD proteins. Each motif is represented by different colored boxes with the serial number located in the center of the box. The colored boxes were ordered manually based on the MEME server results. The length of each colored box does not represent the virtual motif size of the corresponding proteins.

**FIGURE 4:**
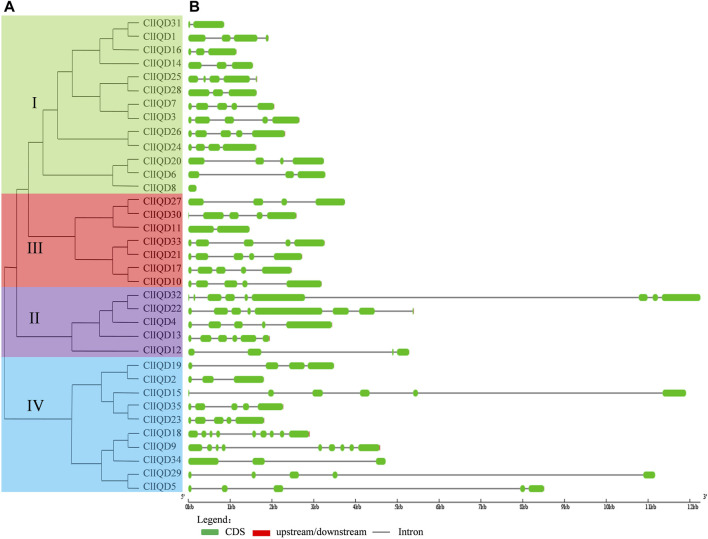
The phylogenetic relationships and exon/intron distributions of the 35 ClIQD genes. **(A)** The unrooted phylogenetic tree generated by the MEGA 7.0 program using the full-length amino acid sequences of the 35 ClIQD proteins. The tree was generated using the maximum-likelihood (ML) method with 1000 bootstrap replicates. **(B)** Exon/intron organization of the 35 ClIQD genes. Exons and introns are represented by green boxes and black lines, respectively.

To comprehensively understand the evolutionary relationships and diversification of watermelon ClIQD proteins, the conserved motifs and exon-intron organization of the 35 ClIQDs were analyzed using MEME online software (https://meme-suite.org/meme/), (Figure 3, Figure 4). There were multiple conserved motifs in the ClIQDs amino acid sequence, squares with different colors represented different conserved motifs (Figure 3; Supplementary Table S2). The conserved motifs were analyzed by SMART (http://smart.embl-heidelberg.de/smart/) and Pfam (http://pfam.xfam.org/search/) online software. Motif 1 and motif 6 were the core sequences of the ClIQD proteins and were widely distributed across the watermelon ClIQD proteins. The other eight motifs with unknown functions were also identified. The proteins with relatively close evolutionary relationships had the same composition motif pattern e. g, ClIQD24/ClIQD26, ClIQD17/ClIQD21 indicating that proteins from the same ClIQD subfamily had functional similarity (Figure 3). Some differences were also detected in the motif composition among different subfamilies. For example, motif 10 was only detected in subfamily IV, which indicated that ClIQD proteins in different subfamilies may serve different functions.

The exon-intron structures of the 35 ClIQD genes in watermelon are shown in Figure 4. There was a structural similarity between the genes with a close evolutionary relationship. The 35 ClIQD genes had length differences and diverse structural types. The number of introns among the 35 ClIQD genes ranged from 0 to 8. The total number of genes with less than or equal to 3 introns was 15, accounting for 42.86% of the 35 ClIQD genes. The total number of genes with less than or equal to 5 introns was 32, accounting for 91.43% of the 35 ClIQD genes. In addition to ClIQD8 with 0 introns, there was an intron detected in the conservative domains of most ClIQD genes.

### Transcript profiles and qRT-PCR verification of the ClIQD genes during watermelon fruit development

**FIGURE 5:**
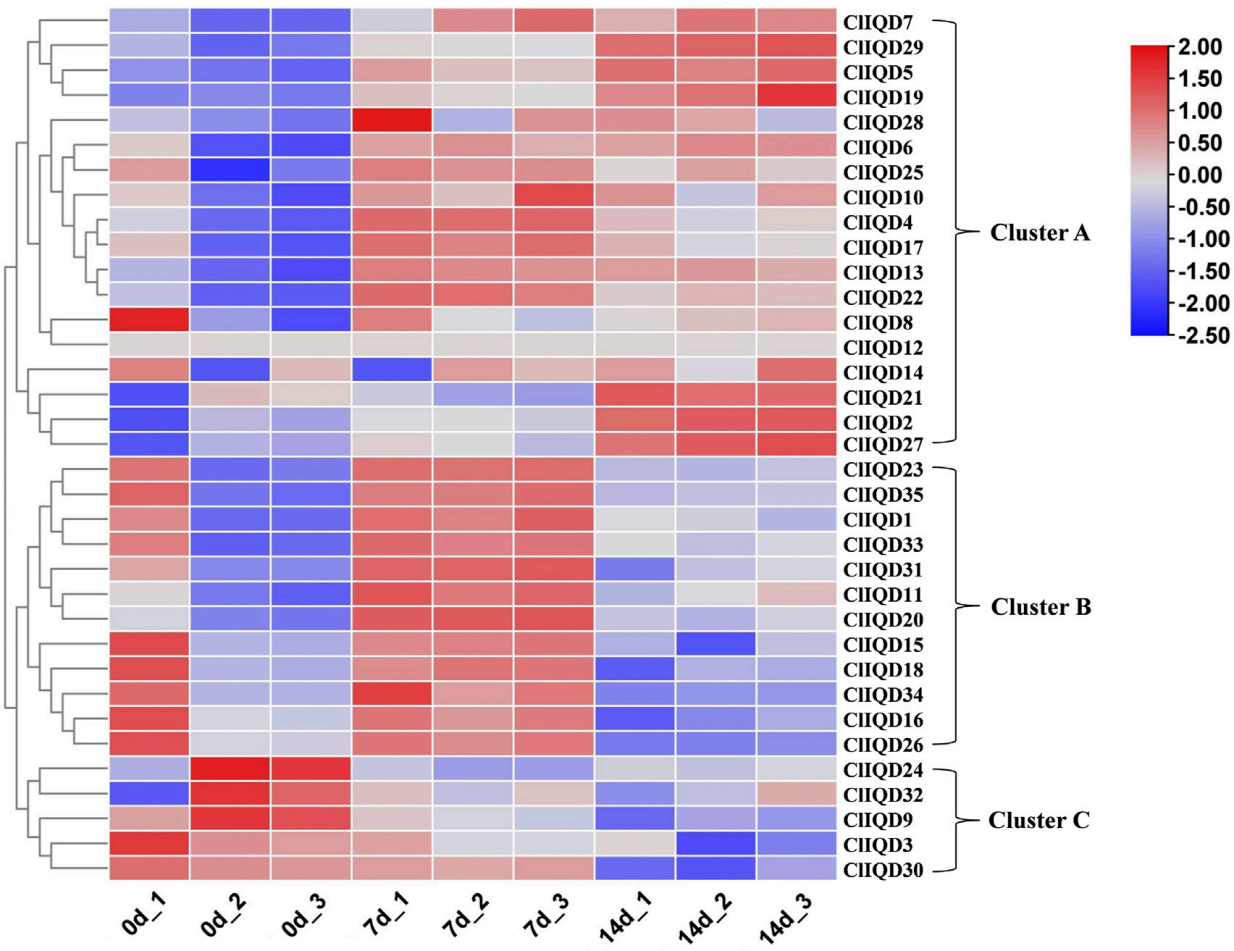
The heatmap analysis of the 35 ClIQD genes during three periods of fruit development (0 days after pollination, 7 days after pollination, 14 days after pollination). Samples were collected during each period in triplicate.

Previous studies have reported on IQD regulation during fruit development (Choi et al., 2005; Xiao et al., 2008; Rodriguez et al., 2011; Cai et al., 2016; Bi et al., 2018; Wu et al., 2018; Yang et al., 2020). To preliminary investigate the functions of ClIQD genes during watermelon fruit development (0 DAP, 7 DAP, 14 DAP), nine RNA-seq libraries were constructed and sequenced from watermelon WM102 rind samples, which included three independent biological replicates. A heat map of 35 watermelon ClIQD genes was constructed using the fragments per kilobase of exon million mapped fragments (FPKM) values from the RNA-seq data to estimate the gene expression levels (Figure 5; Supplementary Table S4). The heat map showed that the 35 watermelon ClIQD genes clustered into three groups. Cluster A contained 18 members of 35 ClIQD genes, which were mainly upregulated during fruit development. Cluster B contained 12 members of 35 ClIQD genes, which were mainly upregulated only at 7 DAP. While Cluster C contained only 5 members of 35 ClIQD genes, which were mainly downregulated during fruit development. Therefore, we concluded that these ClIQD genes may serve different functions in different groups during watermelon fruit development.

**FIGURE 6:**
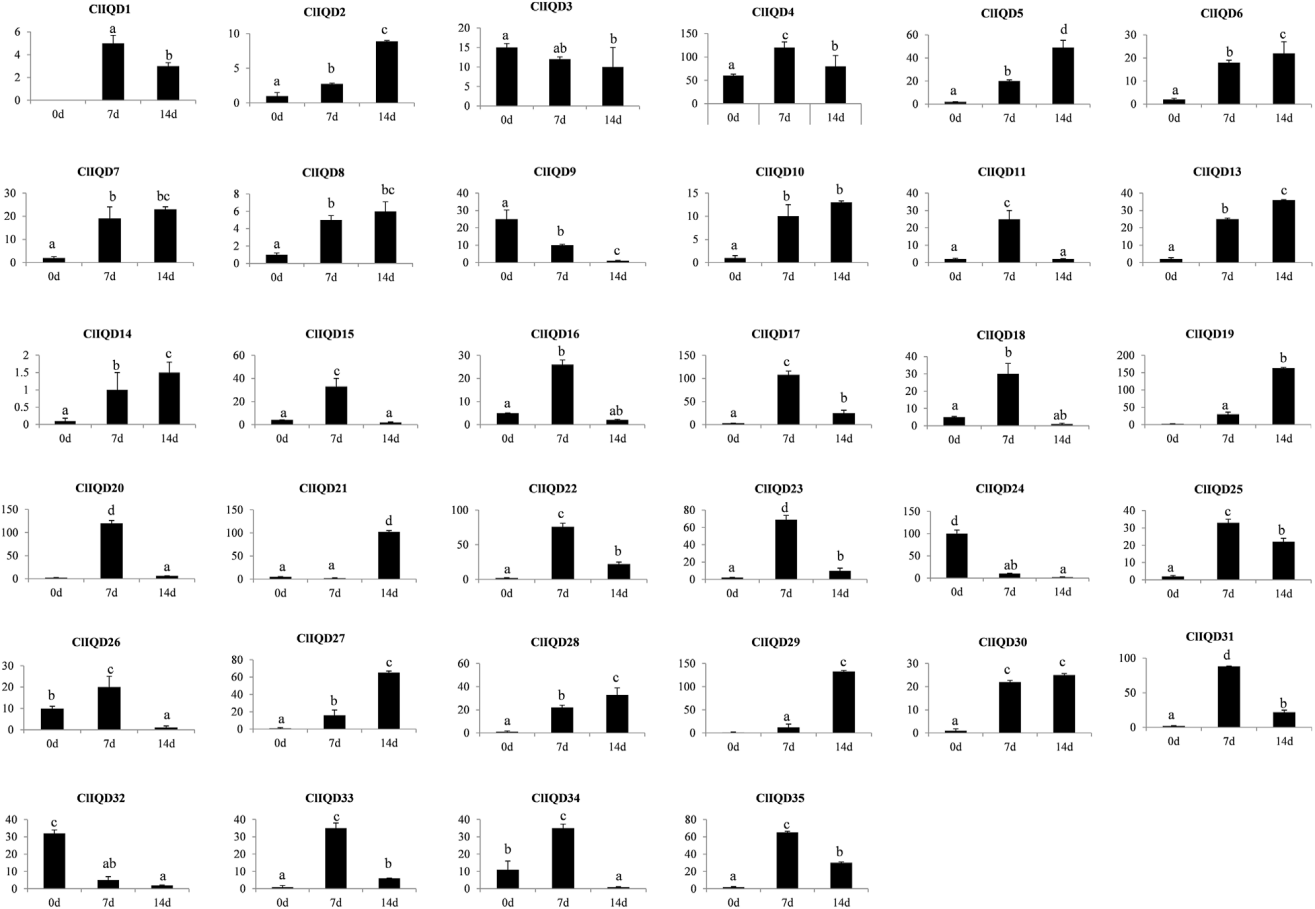
Expression patterns of the 35 ClIQD genes during three periods of fruit development were analyzed by quantitative real-time (qRT-PCR). Error bars represent the standard deviations of three independent replicates. Y-axis represents for expression level.

To verify the RNA-seq results, the fruit rind samples were extracted at 0 DAP, 7 DAP, and 14 DAP and placed in a −80°C freezer for future RNA extraction using three independent biological replicates per time point. The gene expression patterns are shown in Figure 6. ClIQD12 gene was not expressed in each period during fruit development, indicating that it did not function in fruit development. As fruit developed, the expression of three genes (ClIQD9, ClIQD24, ClIQD32) were decreased gradually; the expression of 13 genes (ClIQD2, ClIQD5, ClIQD6, ClIQD7, ClIQD8, ClIQD10, ClIQD13, ClIQD14, ClIQD19, ClIQD27, ClIQD28, ClIQD29, and ClIQD30) were increased gradually and the expression of 16 CIIQD genes (ClIQD1, ClIQD4, ClIQD11, ClIQD15, ClIQD16, ClIQD17, ClIQD18, ClIQD20, ClIQD22, ClIQD23, ClIQD25, ClIQD26, ClIQD31, ClIQD33, ClIQD34 and ClIQD35) was increased at first and then decreased during fruit development. These results are consistent with the RNA-seq results. The expression of ClIQD21 decreased at 0 and 7 DAP and then increased during fruit development. Thus, we concluded that these genes may play important roles during fruit development. Moreover, the highest expression level of ClIQD24 was found only in the ovaries at 0 DAP but had low expression during other periods, which indicated that ClIQD24 may play an important role in ovary development and morphological completion.

### Ectopic expression of ClIQD24 in tomato

**FIGURE 7:**
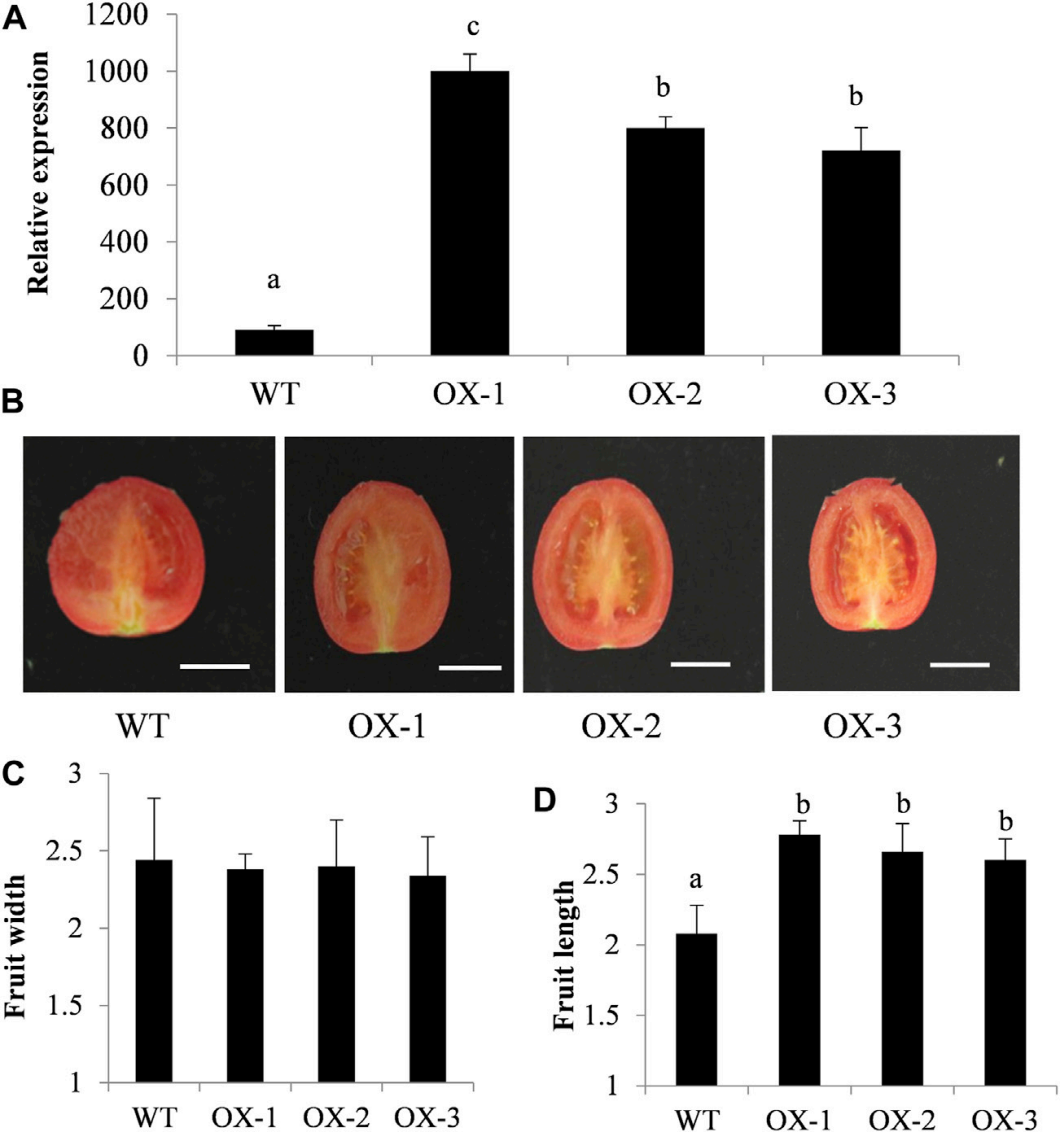
Phenotypic characterization of 35S:ClIQD24 transgenic plants in tomato. **(A)** The expression level of ClIQD24 in the control and three transgenic lines. **(B)** Phenotype of ClIQD24 overexpression in Mic-Tom tomato. **(C-D)** The statistical analysis of fruit width and length (10 individuals were evaluated for each line), the unit of measurement was cm.

ClIQD24 is a homologous gene of the SUN, which was previously reported to regulate fruit shape (Wu et al., 2011; Yang et al., 2020). To verify the function of ClIQD24 in regulating watermelon fruit shape, the overexpression vector, ClIQD24-pRI101, was transformed to tomato Micro-Tom. A total of seven transgenic positive plants were obtained. The expression levels of ClIQD24 in the overexpression (OX) lines greatly increased compared to the control (Figure 7A), indicating that the overexpression vector, ClIQD24-pRI101, was transformed to tomato successfully. The phenotypes of fruit length, width, and shape indices were measured and calculated at the fruit maturity using three overexpression (OX) lines (Figure 7), five fruits from each line were randomly measured. The fruit length of the overexpressed lines increased when compared to the control, but no significant differences were detected in the fruit width between the overexpression lines and control (Figures 7B,C). Our results are consistent with previous studies on other crops (Rodriguez et al., 2011; Bi et al., 2018; Wu et al., 2018), which suggests that ClIQD24 may play an important functional role in regulating watermelon fruit elongation.

### Analysis of miRNA targeting watermelon ClIQD24 gene

**FIGURE 8:**
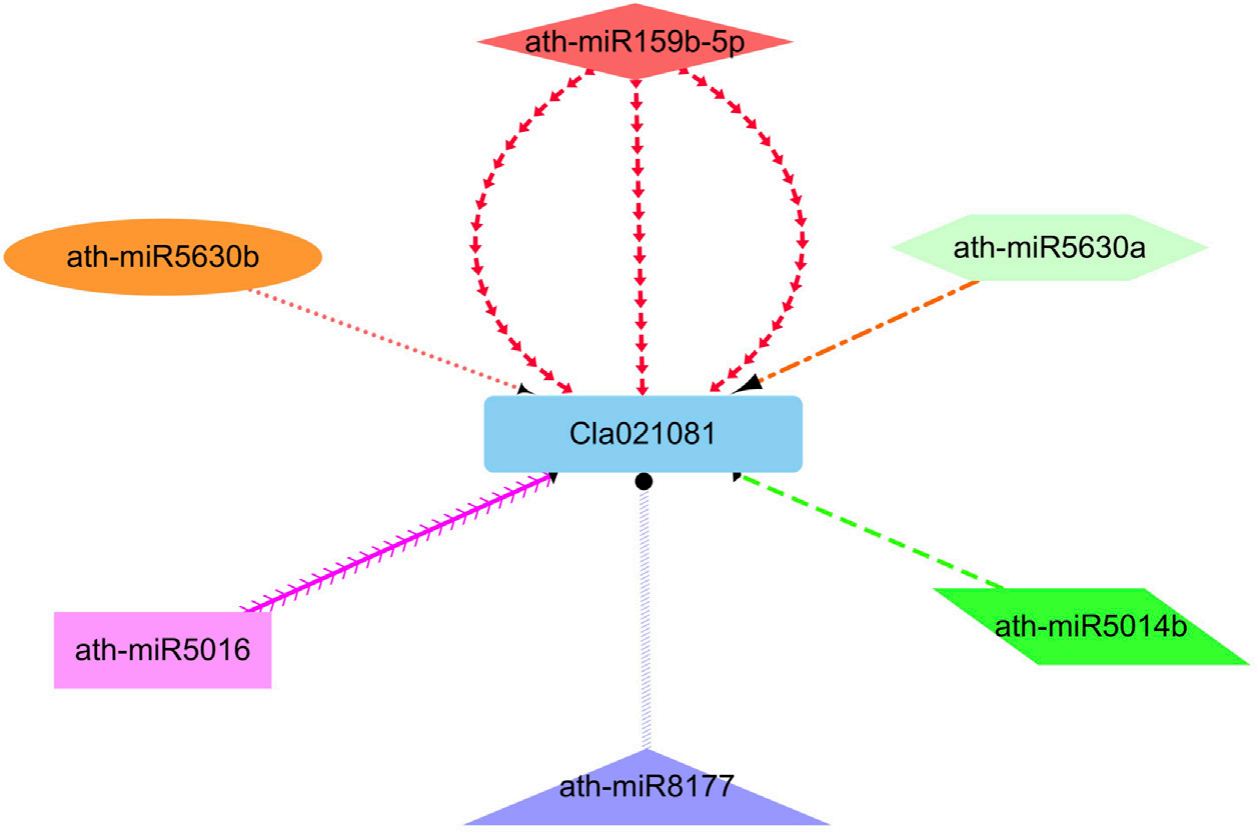
The network representation of the regulatory connections among the predicted miRNAs and ClIQD24 genes. Different colors highlight the interacting miRNAs.

As reported earlier that the miRNAs reliant regulations have a major effect on plant growth as well as regulation. Hence, enhance our knowledge of the miRNAs related regulation of watermelon ClIQD24 gene that participates in the regulation of cell shape and growth, we identified six putative miRNAs (miR159b, miR5630a, miR5630b, miR5014b, miR5016, miR8177) targeting ClIQD24 gene (Figure 8). In-depth details of the miRNA targeted sites are presented in Supplementary Table S7.

## Discussion

The importance of IQD proteins in the regulation of cell shape and growth has been elucidated in various plants (Feng et al., 2014). IQD proteins regulate cell shape and growth through CaM-dependent Ca^2+^ signaling in Arabidopsis (Burstenbinder et al., 2017). Additionally, the overexpression of wheat TaIQD in Arabidopsis caused cotyledons to become narrow and long and affected the spatial arrangement of late pods (Abel et al., 2013). Furthermore, the BFS encoding IQD protein was overexpressed in round fruits when compared to long cylindrical fruits during the ovary formation, thus uncovering the involvement of IQD proteins in the shape of wax gourd fruits (Cheng et al., 2021). However, its functional role in watermelon remains unknown. In our study, 35 IQD genes were identified in watermelon and named ClIQD1- ClIQD35 after a genome-wide analysis. We found that the ClIQD genes were unevenly distributed across the 11 chromosomes, which contained 64–1073 amino acids and had a molecular weight ranging from 7.3 kD to 117.1 kD. Compared to 33 AtIQDs, 28 OsIQDs, and 33 SlSUNs, we found that the number of IQD genes in watermelon was higher. Subsequently, based on the phylogenetic analysis of the IQD genes in watermelon, Arabidopsis, Oryza sativa, and tomato, the ClIQDs were classified into four distinct subfamilies (group I, group II, group III, and group IV), which was supported by the motif and gene structure analyses. Our data indicated that the ClIQD genes in different subfamilies may have different functions.

Recently, IQD genes have been documented to play vital roles in plant development, drought tolerance, and fruit shape (Knaap et al., 2014; Guo et al., 2020). For instance, the tomato IQD gene, SlSUN24, promotes seed germination through ABA signaling (Bi et al., 2018). In addition, SUN was identified to encode a member of the IQD family belonging to CaM-binding proteins that are involved in fruit elongation (Guo et al., 2020). Moreover, deletion of ClFS1 encoding IQD proteins reportedly contributed to the watermelon fruit shape (Dou et al., 2018). To further determine the exact role of IQD proteins in watermelon, we evaluated the expression of ClIQD genes during fruit development. We found that 18 ClIQD genes were mainly upregulated during fruit development, 12 ClIQD genes were mainly upregulated only at 7 DAP, and 5 ClIQD genes were mainly downregulated during fruit development. These findings suggested that ClIQD genes acted as crucial regulators of watermelon fruit development.

To comprehensively investigate the role of ClIQD genes during watermelon fruit shape development, we conducted a qRT-PCR assay to evaluate the expression patterns of 35 ClIQD genes during fruit development. No transcription was detected in the expression of the ClIQD12 gene during each period of fruit development. The expression of ClIQD3, ClIQD9, ClIQD24, and ClIQD32 was decreased gradually during fruit development. The expression of 13 ClIQD genes in Cluster A of RNA-seq was increased gradually during fruit development, and the expression of 16 ClIQD genes in Cluster B of RNA-seq was increased at first and then decreased during later fruit development stages. While the expression of the ClIQD21 gene decreased at first and then increased during fruit development. These data implied that the ClIQD genes may play prominent roles during the development of watermelon fruit shapes.

Since the ClIQD24 gene was only mostly expressed in ovaries period during fruit development, we concluded that ClIQD24 may play an important role during ovary development and morphological completion in watermelon. Therefore, we focused on elucidating the role of ClIQD24 in the development of watermelon fruit shapes. ClIQD24 is a homologous gene of the SUN, which is upregulated during flowering and fruit development, as well as affects the shape of tomato through the regulation of cellular Ca^2+^ signal transmission and cell extension (Clevenger et al., 2015). Herein, through the ectopic expression of ClIQD24 in tomato, we found that the overexpression of ClIQD24 increased the length of tomato, which corroborates the findings of a previous study that ClIQD24 participates in the regulation of cellular elongation (Rodriguez et al., 2011). And ClIQD24 was the potential target gene of miR159 which had been identified to regulate the development of reproductive organs (Hou et al., 2018). All the results revealed that ClIQD24 plays a vital role in the modulation of fruit elongation in watermelon.

MicroRNAs (miRNAs), that are a group of single-stranded, non-coding micro RNAs, are involved in post-transcriptional gene regulation (Cui et al., 2020). Various miRNAs have been identified via genome-wide analysis that are involved in growth and development in plants (Zhang et al., 2007; Kwak et al., 2009; Ayubov et al., 2019). The current study identified six miRNAs belonging to different families (miR159b, miR5630a, miR5630b, miR5014b, miR5016, miR8177) targeting ClIQD24 gene. Discussed miRNAs in the current study are all involved in the plant growth and development as reported earlier (Li and Zhang, 2016; Wang et al., 2017; Wang et al., 2021). These studies suggest that these identified miRNAs might play potential roles in the regulation of cell shape and growth by modifying the transcript level of the ClIQD24 gene in watermelon.

In summary, we elucidated the bioinformatics analysis of 35 IQD genes, including their structures, chromosomal locations, evolutionary divergence, and functions in watermelon. We found that the overexpression of ClIQD24 promoted the fruit elongation in tomato, uncovering the importance of ClIQD24 in the progression of watermelon fruit shape. Our findings lay a foundation for future studies on the molecular mechanism of watermelon fruit shape formation and will serve as a reference for investigations on the fruit shape of other crops.

## Data Availability

The datasets presented in this study can be found in online repositories. The names of the repository/repositories and accession number(s) can be found below: https://www.ncbi.nlm.nih.gov/, PRJNA549842.
